# Supplementary material for: Are carbon emissions trading and green financial instruments synergistic? -Comprehensive quantitative research based on content analysis
Source: PLoS One. 2024 Mar 7;19(3):e0298601. doi: 10.1371/journal.pone.0298601 (PMC10919601; doi:10.1371/journal.pone.0298601)
Supplement: S1 Data — (ZIP) [file pone.0298601.s001.zip › Data/地级市Z_F工作报告.zip!/╝¬┴╓/╝¬┴╓╩╨/└┤╫╘╣1⁄2╓┌║┼í╛┬φ┐╦╩2╛▌═°í┐_2018.docx]

各位代表：

　　我代表市政府向大会报告工作，请予审议，并请政协委员提出意见。

　　一、2017年工作回顾

　　2017年，市政府与全市广大干部群众在市委的坚强领导下，紧密团结在以习近平同志为核心的党中央周围，认真贯彻落实省委省政府各项决策部署，以建设旅游文化名城、新型产业基地、生态宜居城市为目标引领，以构建“6411”产业体系为转型路径，以实施“双十”项目工程为核心支撑，克难攻坚，奋力作为，全面落实年初确定的155项重点任务，经济社会保持了稳中有进、稳中向好的发展态势。地区生产总值预计实现2640亿元，可比增长5%；地方级财政收入105亿元，税收占地方级财政收入比重提高10.5个百分点；城镇和农村常住居民人均可支配收入分别达到27180元、12960元，增长6.5%和5.5%。

　　一年来，主要做了以下工作：

　　（一）突出抓经济运行，稳增长收到实效

　　稳投资。以春季“三早”、秋季“大巡检”等活动为载体，大力实施 “双十”项目工程，集中开展征迁、批办、融资、招商等攻坚行动，全年完成征收751万平方米，开工5000万元以上项目482项，其中亿元以上335项、竣工238项，完成固定资产投资2018.2亿元。大力开展招商引资，比亚迪云轨产业园等一批重点项目签约落地，全年新开工招商引资重点项目202项。稳工业。强化“上争”，为286户企业争取各类专项及奖补资金2亿元；推进银企保对接，净增工业贷款20亿元；建立市级领导包联重点企业制度，为企业解决各类问题460项；开展企业增产增收和减亏增盈攻坚，规模工业利润实现155亿元，增长31%。促消费。培育滑雪+温泉、养老+医疗等新兴消费需求，昌邑万达广场等大型商业综合体竣工运营；电子商务保持强劲发展势头，国家电子商务与物流快递协同发展试点城市建设通过验收，棋盘、黄松甸建成“中国淘宝村”，全市网络零售额同比增长50%。增出口。对外贸易同比增长21%，位列全省第一。

　　（二）突出抓产业升级，调结构步伐加快

　　传统产业改造升级进展顺利。推进化工“补链”，康乃尔化工60万吨甲醇制烯烃一期、博大生化30万吨乙醇扩能等项目相继竣工。推进冶金“转型”，吉林铁合金、吉林炭素正式签订搬迁协议，昊融羰基铁等6个技改项目开工建设。推进汽车“上量”，吉汽龙山整车生产项目竣工投产，森雅系列整车销售突破6万辆。推进农产品加工“提质”，农业产业化龙头企业发展到300户，农产品加工业销售收入增长12%。新兴产业快速发展。温泉产业带综合开发等项目加快建设，四季旅游产品体系日趋完善，旅游总收入增长15%。碳纤维产业基地建设全面展开，应用研发中心开工，大丝束碳纤维生产填补国内空白，中国碳纤维产业大会在我市召开。全国首批通航产业综合示范区试点城市成功获批。医药健康产业6大园区、2大聚集地建设初见成效。现代服务业水平提升。保税物流中心口岸平台功能不断放大，大仝软件服务外包基地等服务业集聚区带动能力增强；双元科技挂牌新三板，吉林华通登陆吉交所，服务业占GDP比重提升到48.7%。现代农业建设稳步推进。万昌、孤店子现代农业先导区示范带动作用明显增强，市级以上现代农业产业园发展到7个，实施农田水利建设项目326项，建成可视化现代农业基地30家，家庭农场、农民专业合作社分别发展到2165个和4456个，无规定疫病区一次性通过国家评估验收，中新食品区成为国家首批农业对外开放合作试验区。

　　（三）突出抓改革创新，强动力有效释放

　　重点领域改革不断深化。推进国资国企改革，组建吉发集团和市投融资担保公司，“三层架构”监管运营模式雏形初现；43户市属国有企业实现集中统一监管。推进市场体系改革，“多证合一”登记制度全面推行，市场主体全程电子化登记工作经验在全省推广。非居民用天然气、交通、污水等相关价格改革取得阶段性成果。售电侧改革取得实质性进展，国兴售电公司挂牌成立。不动产统一登记制度全面落实。推进社会领域改革，城乡义务教育一体化改革和技术技能人才创新培养改革启动实施，公立医疗机构综合改革和分级诊疗制度全面推开。推进农村综合改革，农村土地承包经营权确权完成市县两级成果验收，土地收益保证贷款、经营权抵押贷款累计发放5.5亿元，79家国有林场完成年度改革任务，集体林权交易流转1.2万亩。创新驱动战略深入实施。细分行业应用技术研究院发展到24家，22项校企合作项目加速实施，产学研用协同创新迸发新的活力。校城融合“双创”基地工作被国务院作为全省唯一典型通报表扬，大众创业、万众创新的社会氛围更加浓厚，新增“四上”企业299户，民营经济占地方级财政收入比重提升到79%。

　　（四）突出抓共享发展，惠民生成果丰硕

　　脱贫攻坚取得阶段性成果。持续开展精准脱贫春季、秋季攻势，以光伏发电为重点的产业扶贫实现贫困村全覆盖，超额完成贫困户危房改造任务，183项安全饮水工程完工，46个省级贫困村出列，1.11万名建档立卡贫困人口实现脱贫。生态环境建设不断加强。全力配合中央环保督察，深入实施蓝天、碧水等工程，秸秆禁烧、燃煤锅炉淘汰整治等大气污染防治措施持续强化；农村“六清”人居环境整治成效显著；河长制全面建立，“九河一湖”黑臭水体等专项整治集中开展，市污水处理厂提标改造工程投入运行。社会保障体系不断完善。在全省率先完成农村低保补差式救助工作，城市、农村低保标准分别提高到每人每月516元和每人每年3685元；新农合最高补偿额度提高到20万元；企业退休人员养老金实现13连调。改造城市棚户区7326套，分配公租房4039套，发放住房租赁补贴8697户，完成77个老旧小区宜居改造和1585户农村危房改造任务。社会事业蓬勃发展。义务教育大学区制全面推行，成功承办全国中学生田径联赛、全国职业院校技能大赛并获得佳绩；以市级综合医院和专科医院为核心的多层次医疗联合体建成；国家扩大文化消费试点城市工作全面启动，全国影视指定拍摄景地正式获批；市冰球队组建并出征俄罗斯超级冰球联赛，冰雪奥运冠军培育计划大力实施，获得国家级以上赛事金牌70枚；高水平举办雪博会暨雾凇冰雪节、第二届国际马拉松等大型活动，城市美誉度和影响力显著提升。健全安全生产长效机制，认真落实领导干部包案和接访下访制度，深入开展社会治安综合治理，安全和谐稳定的社会局面持续巩固。

　　（五）突出抓“放管服”改革，政府建设全面提速

　　深入落实国务院和省政府取消下放行政审批事项，387项行政许可项目纳入政务服务中心规范运行，清理取消职业资格许可认定事项433项。承担行政职能事业单位改革试点任务较好完成。在全省率先建成政务云平台，公共资源交易开启“阳光模式”，网上政府信息公开平台运行良好。投资项目大数据服务平台上线试运行。

　　去年，我市连续遭遇“7·13”“7·19”两次特大洪涝灾害，袭击范围之广，破坏力之强，突破历史极值。我们认真贯彻落实习近平总书记批示指示精神，在省委省政府的坚强领导下，以保护人民群众生命安全为首要任务，众志成城，抗洪抢险，累计转移安置群众50余万人次，迅速恢复供水、供电、通讯、交通等设施功能，灾区群众基本生活生产需求得到及时有效保障。市委市政府被评为全省抗洪抢险突出贡献集体。灾后第一时间出台重建政策措施20个，争取到国家和省灾后应急项目及生活救助等各类资金近8亿元，900项年度重建任务全面完成，特别是12374户重建和修缮房屋全部完工，切实保障了受灾群众温暖过冬。

　　各位代表，过去一年，我们在复杂外部环境面前负重前行，在严峻困难挑战面前砥砺奋进，取得了极为不易的发展成果，这是我们认真贯彻党中央各项决策部署的结果，是省委省政府、市委坚强领导的结果，是各县（市）区、各部门和全市广大干部群众团结奋进的结果，凝聚了各方面的心血和智慧。在此，我代表市政府，向全市各族人民，向人大代表、政协委员、各民主党派、工商联和社会各界人士，向解放军驻吉部队、武警官兵和公安干警，向中省直各单位以及所有关心、支持吉林市新一轮振兴发展的海内外朋友，表示衷心的感谢并致以崇高的敬意！

　　同时，我们也清醒地认识到面临的问题：发展不平衡、不充分的矛盾比较突出，经济下行压力仍然较大，产业转型升级任重道远；一些重点领域改革有待更大突破，制约发展的体制机制障碍尚未有效破解；财政收支矛盾加剧，公共服务供给与群众期盼还有一定差距；政府效能亟待提高，营商环境有待进一步优化。对于这些问题，我们一定以高度负责的精神，认真加以解决。

　　二、2018年工作安排

　　今年是全面贯彻落实党的十九大精神的开局之年，是进入新时代、站在新起点、决胜全面建成小康社会、建设幸福美好吉林的关键之年。政府工作的总体要求是：高举习近平新时代中国特色社会主义思想伟大旗帜，全面贯彻党的十九大精神，认真落实省委第十一次党代会、十一届二次全会部署和市委十三届三次全会的总体安排，坚持稳中求进工作总基调，深入贯彻新发展理念，按照高质量发展的要求，立足城市发展定位，以“五谋五建”为统领，实施五大发展举措，打好三大攻坚战，强化一大载体支撑，向着决胜全面小康、建设幸福美好吉林目标不懈奋斗，全力开创老工业基地振兴发展的新局面。

　　做好今年工作，必须坚持以下原则：

　　第一，坚定不移高举习近平新时代中国特色社会主义思想伟大旗帜。坚持用习近平新时代中国特色社会主义思想武装头脑、指导实践、推动工作，牢固树立“四个意识”，坚决维护以习近平同志为核心的党中央权威和集中统一领导，在“四学四转”上下功夫，奋力开创新时代吉林市全面振兴发展的新局面。

　　第二，坚定不移贯彻落实新发展理念。牢牢抓住发展这个第一要务，把创新、协调、绿色、开放、共享五大发展理念贯穿于经济社会发展全过程和各领域，加快建设动力之城、特色之城、生态之城、活力之城、和谐之城，努力走出一条更高质量、更有效率、更加公平、更可持续的振兴发展新路。

　　第三，坚定不移建设现代化经济体系。始终坚持质量第一、效益优先，以供给侧结构性改革为主线，大力构建“6411”产业体系，推进城乡统筹发展，加快改革开放步伐，滚动实施“双十”项目工程，推动经济发展质量变革、效益变革、动力变革，不断增强经济发展的创新力和竞争力。

　　第四，坚定不移落实以人民为中心的发展思想。把人民利益摆在至高无上的地位，坚持尽力而为、量力而行，深入开展脱贫攻坚，推进社会事业协调发展，完善公共服务体系，在发展中补齐民生短板、促进社会公平正义，不断满足人民日益增长的美好生活需要。

　　第五，坚定不移改善发展环境。把改善发展环境作为构筑竞争优势的战略任务，不断深化行政审批制度改革，健全完善投资创业政策体系，全面抓好“诚信吉林”建设，持续规范市场经济秩序，全力营造优质高效的政务环境、公平竞争的市场环境、公平公正的法治环境。

　　主要预期目标是：地区生产总值增长6%左右，地方级财政收入可比增长5%，城镇和农村常住居民人均可支配收入与经济增长保持同步，城镇新增就业7万人，城镇登记失业率控制在4.5%以内，单位GDP能耗下降2%以上。在实际工作中，我们要坚持稳中求进工作总基调，努力争取更好结果。

　　（一）实施五大发展举措

　　1.着力建设现代产业体系。我市新一轮振兴发展，必须立足现有产业基础，以实体经济为着力点，深化供给侧结构性改革，持续发力、久久为功，建设实体经济、科技创新、现代金融、人力资源协同发展的产业体系。

　　做强做优实体经济。坚持高质量发展的要求，处理好传统产业转型升级和新兴产业发展壮大的关系，稳中求进，实现动力转换、效率提高、质量提升。培育壮大新兴产业。利用好近几年冰雪产业强劲的发展势头，以冰雪、温泉等为特色，率先在旅游产业取得新突破，力争旅游收入突破千亿元。大力实施冰雪产业升级工程，组织实施北大壶冰雪体育旅游产业升级改造项目，开工建设东山冰上运动核心圈、圣德温泉小镇等12个重点项目，推动北山四季越野滑雪场竣工运营，积极创建松花湖5A级景区。深挖历史文化名城内涵，推进文化与旅游融合发展，打造一批独具地域特色的文化旅游产品，全年接待游客同比增长15%。壮大碳纤维产业集群，继续实施吉林碳谷4万吨碳纤维原丝、精功8000吨大丝束碳纤维等项目，增加原丝、碳丝产能，同时精准引进碳纤维下游企业，着力打造“中国碳谷”。规划建设通航产业示范区、生物制药产业园、华微电子半导体产业园等园区，加速新兴产业集聚化发展。推动互联网、人工智能、大数据等与实体经济融合发展，引进战略投资者，推进京东集团以无人机制造为核心的产业项目，加快培育形成新的产业。改造提升传统产业。克服吉化检修因素影响，全力推进工业稳定增长。加快化工产业向精细化、高端化、绿色化发展，重点抓好化工四大产业链条延伸，实施汽油国Ⅵ质量升级烷基化、12万吨重芳烃轻质化等重点项目；建设燃料乙醇生产基地，力争新增产能90万吨。支持一汽吉林汽车、吉汽龙山汽车持续释放产能，推进万丰工业园二期等核心零部件企业建设，用好通田汽车资质，引进新能源汽车生产企业。促进冶金产业转型升级，加快吉铁、吉炭搬迁改造及新厂区4万吨石墨电极、6.5万吨特种合金生产项目建设步伐，规划建设北方小五金生产基地。积极发展智能制造，推进工业化和信息化深度融合。加快汽车、化工等八大行业两化融合，开展数字化车间等智能制造新模式推广应用，谋划焊接机器人、智能化物流设施等项目，国家两化融合试点企业达到12户。推进质量强市建设，增加中高端产品有效供给，年内新增省级以上名牌产品20个。规模工业增加值增长4%左右。加快发展现代服务业。支持恒涛锅炉、东杰科技等制造业服务化企业发展壮大，争创全省首批制造业与服务业融合发展试点城市；引进培育龙头电商，扶持本土电商企业快速成长，推进电子商务进农村；培育发展新业态、新模式，开展文化消费改革试点，发展健康养老等产业，完成河南街传统商圈改造，改善东市场消费环境，开展“放心消费在江城”创建活动。社会消费品零售总额增长8%。

　　加快科技创新步伐。创新是实体经济发展的第一动力，我市要用好校城融合、企业家论坛等机制，加强创新体系建设，加快科技成果转化，深化科技体制改革，逐步实现实体经济发展的动力转化。推动科技与产业发展融合。提高科技与我市特色产业的关联度，围绕新材料、新能源等新兴产业，推动碳纤维研究院、生物质炼制研究院等优势创新平台建设。推动高校、科研院所、企业、创客空间等创新主体融合。建设东北电力大学科技产业园等六大创新创业基地，支持高校、科研院所、大型企业建设行业性、专业化创客空间和企业孵化器，形成创新型产业链生态体系。新培育国家高新技术和科技“小巨人”企业各30户，科技成果本地转化20项以上。推动产学研用融合。支持化工学院与吉林化纤建立联合实验室，加快吉林大学吉林市研究院、中科院应化所中试基地建设，发挥中科院、浙大等在吉技术转移中心和百度创新中心载体作用，深化北华大学等高校与国外科研机构开展国际交流合作。推动人才、资本、信息与技术等创新要素融合。建立离岸人才孵化器，发挥国家科技领军人才创新驱动中心等平台作用，支持“三高”人才回归创新创业。放大科技创业投资基金、科技风险补偿基金等使用效能，大力发展科技金融服务机构，争创全省科技金融示范基地。充分挖掘运用信息资源，布局打造吉林浙达能源清洁化、大型仪器共享服务平台等一批行业性、专业化“双创”大数据云平台，市科创中心投入运营。

　　强化现代金融支撑。充分用好现代金融工具，让更多金融资源投向实体经济。深化产融合作。抓好国家产融合作试点城市建设，搭建产融合作服务平台，建立融资需求“红名单”，设立企业应急周转金，提升金融服务制造业的水平。创新财税金融支持方式。发挥财税“四两拨千斤”的作用，支持银行、保险公司和融资担保机构大力发展普惠金融，降低企业融资成本。加强政银企合作，拓展“种子基金”“助保金池”功能，探索供应链金融合作模式，提高金融供给水平。发挥产业基金引导作用。充分用好现有产业基金，设立新能源、碳纤维、医药健康等产业子基金，撬动社会资本35亿元，促进战略性、基础性、先导性产业加快发展。发挥资本市场融资功能。抓住国家对符合条件的东北地区企业IPO优先支持的政策机遇，启动企业上市工程，年内力争2户企业上市、8户企业挂牌，再培育20户备选企业。积极利用债券市场，重点培育信用等级高的发债主体，稳妥利用债券创新品种。加快昌邑金融集聚区建设。实施“一街、一厦、一中心”等重点项目，创新金融产品和平台模式，丰富金融服务业构成，进一步增强承载、集聚、辐射功能。

　　丰富人力资源供给。加大企业家、高级管理人才、职业工人等各层级人才的培养力度，丰富实体经济的人力资源供给。实施企业家素质提升工程。选派企业优秀管理人员到央企及发达地区国企挂职锻炼，依托国内顶尖培训机构或知名高校开展专题培训，组织赴江浙地区开展交流活动。实施人才招引工程。突出“高精尖缺”，加大技术创新专家型人才、高级经济管理人才引进力度，建设引智示范基地和人才小镇，精准引进高端领军人才10人、创新团队10个，新建院士工作站2个。实施大学生本地就业工程。建立高校毕业生创业贷款风险补偿基金，完善大学生就业创业补贴政策，搭建就业双选对接平台，驻吉高校毕业生本地就业率提高到15%以上。实施“江城工匠”培树工程。大力弘扬“工匠精神”， 培养造就一批高技能、知识型、创新型 “江城工匠”，组建高技能人才库，筹建技能大师工作室，培训新技师1000人。完成职教园区搬迁并与产业发展相融合，举办“技兴江城”职业技能大赛，争创国家级高技能人才培训基地。

　　2.着力实施乡村振兴战略。把解决好“三农”问题作为重中之重，按照产业兴旺、生态宜居、乡风文明、治理有效、生活富裕的总要求，率先在全省实现农业农村现代化。

　　建设转型升级新农业。优化产业体系。完成1万公顷高标准农田建设任务，绿色有机水稻种植面积比重提高到37%。新增林下中草药、经济林等林业产业基地12万亩，新建棚室1300公顷，创建6个省级以上畜禽标准化示范场。无规定疫病区保持规范运行。规划实施鑫达农牧科技等重点农业产业化项目10个，农产品加工业销售收入增长12%。休闲农业星级企业突破110户。巩固生产体系。集成推广30项农机农业生产技术，农机化综合作业水平达到78%。扩大可视农业联盟规模，可视化基地达到35个。实施农田水利项目347项。激活经营体系。新培育家庭农场和农民专业合作社各200个，土地流转面积达到14万公顷。扩大“寒地、黑土、天蓝、水绿”农产品区域公用品牌影响力，再认定推广一批“三品一标”农产品。

　　打造美丽宜居新农村。实施农村人居环境整治行动，以52个省级新农村建设为重点，推进吉长南线等7个重点片区联动建设。实施“三线下地”工程，全面扫除乡镇“蜘蛛网”；推进农村垃圾处理，农村地埋式环保桶建设实现重点村屯全覆盖；高标准绿化美化村屯312个、新改建和绿化美化农村公路1300公里，创建省级美丽乡村10个以上，打造美丽庭院、干净人家1.5万户。

　　培育生活小康新农民。壮大新型职业农民队伍。完善技能培训机制，培育一批新型职业农民和职业经理人；加强农业科技服务，培育种植大户、科技示范户4万户，培训各类人员10万人次。拓宽农民就业增收渠道。深入实施农民增收行动，支持高校毕业生、退役军人、科技人员到农村施展才华，扶持创业者2000人，引导农民工等返乡创业1000人以上；培育劳务输出品牌，实现农村劳动力异地转移就业55万人次。

　　3.着力推进区域协调发展。按照建设现代化大城市发展方向，推动中心城区、县域和小城镇协调发展，切实增强区域发展的协同性、联动性、整体性。

　　提升中心城区功能品质。启动新一轮城市总规编制，开展城市“双修”和城市设计，严格执行松花江两岸城市规划，实现中心城区控规全覆盖。新建续建城建重点工程100项，计划投资100亿元。建成晓光路、解放西路与长春路交汇下穿隧道等8项路桥工程，维修武汉路等11条主次街路，畅通微循环。推进安全应急备用水源和六水厂建设；加快实施“一城一网”联网工程，改造老旧供热管网110公里。抓好哈达湾海绵城市试点建设。编制实施新型智慧城市建设总体规划，以智慧城市建设带动大数据、云计算产业发展，争创国家新型智慧城市试点。坚持建管并重的原则，推进城市管理执法体制改革，建立双轨制考核、城警联动、公众参与的运行机制，不断提升城市管理精细化、网格化、常态化水平。深入开展文明城市创建活动，全面提升市民文明素质和社会文明程度。

　　扎实推动县域发展。县域是振兴发展的潜力所在。要立足资源禀赋和产业基础，推进县域和中心城区错位互补、协同并进。永吉要构建“一脊两翼”发展格局，争做全省现代农业排头兵，加快与吉林市同城化发展进程。舒兰要突出抓好国家农业可持续发展试验示范区建设，打造现代农业生态市。磐石要壮大金属冶炼等主导产业，培育 “三大集散地”，建设省内重要医药健康产业基地、东北知名新型金属材料产业基地和新型工业化示范城市。蛟河要着力培育医药健康、新材料、旅游商贸、长白山特产加工等产业动力源，努力实现经济质量、效益和速度的同步提升。桦甸要做优做强绿色食品、医药健康、新能源等新兴产业，提升“山水桦甸·五彩金城”旅游品牌影响力，打造新型能源强市、健康产业基地、生态旅游新城。

　　培育特色小（城）镇。以乌拉街国家级特色小镇、孤店子等省级示范城镇以及庆岭冰酒、北药健康等一批特色小（城）镇建设为重点，争取国家发改委和光大银行千亿级特色小镇基金支持，引进战略投资者，推进小城镇生产、生活、生态融合发展，打造一批全国、全省知名的特色产业小镇。

　　4.着力深化改革开放。改革开放是我市新一轮振兴发展的活力之源。要以改革的勇气破解制约我市发展的体制机制障碍，以开放的胸怀增添我市国际化城市的色彩。

　　 全面深化重点领域改革。深化国资国企改革。强化国有资产集中统一监管，明晰市属国有企业功能定位，改组组建投资运营公司，推进混合所有制改革。制定监管事项清单和投资负面清单，建立国有企业资本市场化补充机制。发挥吉发集团作用，重组整合、创新发展一批市属国有企业。加快剥离42户国有企业办社会职能，完成“三供一业”分离移交和市属国有企业公司制改革任务。深化农村综合改革。巩固农村土地承包经营权确权成果，稳步推进农村集体产权、国有林场、农垦、供销社等改革，推动农村金融产品和服务方式创新，增强农村发展内生动力。支持民营经济大发展快发展。探索建立市场准入负面清单制度，进一步向民营经济开放投资领域。创新服务民营企业方式方法，建立产业联盟，落实好联系民营企业制度，出台管用的加快民营经济发展的政策措施。激发和保护企业家精神，实施企业梯次培育计划，推动民营经济新一轮腾飞。新增“四上”企业190户，“专精特新”企业100户，民营经济主营业务收入增长7%。

　　全方位扩大对外开放。强化开放载体建设。发挥中新食品区、保税物流中心等开放平台作用，推进中德工业园等新兴开放载体建设，加强与世界500强等大企业合资合作，全面提升我市经济对外开放度。加快对外通道建设。深入实施长吉图战略，加快与长春互联互通和产业融合发展步伐，主动参与中蒙俄经济走廊建设，支持化工、纺织、食品加工业等重点企业参与俄罗斯纳霍德卡港口等区域开发，打造省域开放新高地。推动开发区改革创新。加快龙潭等8个经济开发区整合步伐，推动松花湖、航空产业园等申报设立省级开发区，构建“14+3”发展框架；统筹规划开发区产业布局，理顺运行体制和管理机制，改革人事制度，实施差异化绩效管理，激发开发区发展活力，争创全省开发区改革试点。

　　5.着力保障和改善民生。民生是政府一切工作的出发点和落脚点。围绕人民群众最关心最迫切解决的问题，尽力而为，量力而行，扎实办好八方面27件民生实事。优先发展教育事业。开展城区小学生课后免费托管服务，帮助家长解决“接送愁”和“管护难”问题；提升幼托服务质量，完善各类幼儿园建设布局；落实专项资金4500万元，资助城区家庭困难学生，不让一名孩子因贫失学；投入资金1.37亿元，新建、改扩建农村义务教育薄弱学校37所；建成全市共享教育数据中心，启动“吉教云课”工程，开设名师在线直播课程2000节以上。推动就业创业。开展“15分钟就业服务圈”活动，发放创业担保贷款1.7亿元，城镇新增就业7万人。构建健康服务体系。基本公共卫生服务项目由46项增加到55项，深入开展爱国卫生运动，提升居民健康服务保障水平；加快医联体建设，县域内就诊率达到90%以上；家庭医生签约服务实现贫困人口全覆盖。完善社会保障体系。降低城镇职工大型医用设备检查治疗费用和普通住院床位费个人负担，低保困难家庭患者在定点医院开展核磁共振、CT检查减免70%费用，提高参保职工报销比例，全面实现跨省异地就医联网即时结算；为残疾人开展5类33项精准康复服务；开展养老院服务质量提升专项行动，养老机构医养结合率达到80%以上。改善居住环境。加快建立多主体供应、多渠道保障、租购并举的住房制度，完成棚户区改造5702户，回迁安置3000户，发放租赁补贴7000户，对48个老旧小区进行宜居综合改造；加快居民住房产权办理工作；建立物业服务价格形成机制，引入市场化手段规范物业服务市场；改造农村危房1196户、农村厕所62200户。加强交通管理。科学编制停车场规划，改扩建停车场52个，公共停车区域全部施划泊位；采取整治交通违法行为、规范停车收费、清理“僵尸车”等措施，改善中心医院等人员聚集区静态交通秩序；大力发展公共交通，新建高新北区等2个公交场站，调整新开公交线路12条。推进灾区重建振兴。全面完成灾后重建工作任务，谋划实施市政、交通、农林水利、社会事业等领域重大项目；规划建设小牤牛河、远大河流域防洪体系，提升城市防洪功能；编制实施永吉县防洪规划，同步启动永吉县城总体规划修编和给排水工程等其他专项规划编制。丰富群众文体生活。免费开放全市公共文化设施；启动广电传媒中心等项目建设，玄天岭文化景观项目竣工；开展市民文化节、全民阅读推广等文化惠民活动，组织艺术演出150场以上；高水平举办首届世界冰上龙舟赛、全国马拉松锦标赛等赛事活动；开展“全民健身日”系列活动，组织大型体育活动100项（次）以上。

　　巩固社会安定局面。统筹推进安全生产领域改革，压实安全生产责任，推进安全监管规范化制度化建设，突出抓好重点领域安全隐患排查整治，坚决防止重特大安全事故发生。实施放心工程、餐饮服务质量提升工程建设，全面落实“四个最严”要求，切实保障食品药品安全。完善立体化信息化社会治安防控体系，依法严厉打击各种违法犯罪活动，全力维护公共安全；加强信访法治化建设，加大“事要解决”力度，依法维护群众合法权益。

　　（二）打好三大攻坚战

　　打好三大攻坚战是党中央着眼决胜全面建成小康社会作出的重大战略部署。这三方面工作是政府必须完成的政治任务，不能出任何闪失。

　　打好防范化解重大风险攻坚战。今年，尽管我市各类风险总体可控，但对局部风险特别是地方债务风险、金融风险、养老保险支付风险，必须做到早识别、早预警、早发现、早处置。加强政府债务管理。面对经济社会发展对政府净投入的需求与政府有限的举债空间之间的突出矛盾，坚持量入为出的原则，对存量债务，明确责任，加大处置化解力度，健全风险预警、评估、应急机制，坚决防止出现偿债危机。对新增债务，严格管控，坚持限额管理和预算管理。实施PPP项目综合平衡和动态管理；推动平台公司转型，完善治理结构，打造AAA级平台体系，按照市场化原则，支持平台公司自主开展经营活动。有效防范金融风险。探索推进地方金融监管体制改革，严厉打击非法集资活动，加强互联网金融管理。采取多种方式，解决我市部分行业企业杠杆率偏高问题，防止债务风险恶化。引导企业走市场化之路，合理控制投资冲动。金融机构不能简单使用抽、停贷等手段，恶化自身发展环境。坚决防控养老保险支付风险。我市养老保险抚养比偏低，硬缺口已经显现。要积极扩大就业，出台政策挖掘扩面潜力，争取省级调剂支持，多措并举，确保按时足额发放。

　　打好精准脱贫攻坚战。脱贫攻坚已进入“深水区”。要落实“六个精准”要求，完成42个贫困村和1万贫困人口脱贫的年度任务。落实“四个理清”责任机制，发挥数据平台作用，强化项目管理和资金管理。把牢“识别关”，把好“退出关”，确保“三率一度”达到规定标准。以光伏和秸秆综合利用产业为重点，采取多种形式，提升产业扶贫效益。开展“健康扶贫年”“教育扶贫年”系列专项行业扶贫行动，做到政策优先落实、资金优先使用、项目优先安排、工作优先衔接。注重把扶贫同扶志、扶智相结合，提高脱贫对象自我发展能力和脱贫致富内在动力。抓好省扶贫巡视问题整改，加强“插花村”贫困户脱贫工作，强化扶贫资金管理和审计监督，开展扶贫领域作风专项整治，搞好减贫任务检查验收，确保扶贫成果经得起历史和实践检验。

　　打好污染防治攻坚战。优美环境是人民群众美好生活的重要组成部分。针对中央环保督察反馈的问题，以高度的责任感狠抓整改落实，坚决完成好年度任务。实施清洁空气行动。建设普惠新能源光伏发电、70个村级公司秸秆能源化利用等新能源产业项目，建成集中规范的清洁煤炭交易市场、配送中心和销售网络，严格工业企业、机动车等污染源稳定达标排放和减排措施管理，推进燃煤热电机组超低排放改造和燃煤锅炉淘汰整治，重拳治理餐饮业油烟污染。空气质量好于二级以上天数达到280天以上。实施清洁水体行动。深入落实河长制，治理中小河流96条。加强饮用水源地、江河源头保护，推进挡石河、细鳞河等水生态环保项目，实施环松花湖乡镇村屯污水和垃圾收集处理工程，完成小沙河、石井沟河、鸡冠山河等黑臭水体整治任务。实施清洁土壤行动。划定全市生态保护红线面积，建立重点监管企业名单、污染地块目录及开发利用负面清单，加强农业面源污染防治，推进吉铁、吉炭等污染场地生态修复，清收还林10万亩。构建环境治理体系。实行最严格生态环境保护制度，建立环评管“准入”、排污许可管“运行”、监察执法管“落实”的一证式管理模式，以零容忍态度严厉打击生态环境违法行为。

　　（三）强化一大载体支撑

　　一分部署，九分落实。今年政府工作要以项目建设为载体，推动各项重点工作落到实处。

　　完善项目管理大数据平台建设。将政府投资重点项目、招商引资重点项目以及“上争”类项目全部纳入平台动态管理，明确任务清单、进度清单、责任清单，对项目从签约、前期手续、开工建设到投产达效进行全过程跟踪监管，做到随时掌握进度、随时发现问题、及时解决问题，全力推动项目建设取得新突破。

　　滚动实施“双十”项目工程。动态调整“双十”项目工程包，开工子项目135项，完成投资220亿元。完善重大项目（工程）“六个一”包保责任机制、协调服务机制、调度督查机制和考核奖励机制，推动重大项目建设不断取得新突破。

　　大力开展“招商季”行动。变“冬闲”为“冬忙”，市级领导要以上率下，带头走出去招商，带头推动项目落地，组织好赴北京、珠三角等境内招商活动，加大德国、日本、新加坡等境外招商力度，深化与浙江对口合作，在全市形成抢抓项目建设的浓厚氛围。在年初储备1400个重点项目基础上，推动一批续建项目和新签约项目开工建设，推动一批正在洽谈项目签订协议，推动一批“上争”项目得到批准，推动一批谋划项目找到合作伙伴；按照“追新、选优、谋大、图强”的要求，再充实一批项目纳入储备库，力争取得更多务实成果。

　　三、加快建设人民满意的服务型政府

　　打铁必须自身硬。政府全体工作人员必须恪守为民务实清廉本色，牢固树立宗旨意识，持之以恒转变作风，不断提高企业、群众的满意度，切实增强城市的软实力。

　　强化环境建设。对标国内先进地区，以推进“放管服”改革为主要抓手，全面改善服务质量，提高办事效率，加大涉软案件查处力度，全力打造零障碍、低成本、高效率的一流营商环境。做到更加彻底地“放”，精简行政审批事项，完善并联容缺承诺审批机制，探索一枚印章管审批模式，“多证合一”完成率达到100%。实现大厅之外无审批、清单之外无审批。做到更加有效地“管”，创新完善监管模式，“双随机一公开”行政执法业务实现全覆盖；做到更加优质地“服”，加快政务大厅标准化建设，大幅提升实体政务大厅服务能力；推进网上政务大厅建设，推动行政审批事项和公共服务事项上网运行，促进线上线下服务实时同步、实体大厅和网上大厅融合互补，努力实现让企业和群众“办事不见面”“最多跑一次”的目标。

　　强化法治建设。深入推进法治政府建设，切实把政府各项工作纳入到法治化轨道。自觉接受市人大及其常委会法律监督、工作监督和市政协民主监督，主动接受社会监督。深入推进依法行政，落实行政处罚自由裁量基准制度，促进严格规范公正文明执法。发挥市决咨委智囊作用，提高政府科学决策水平。全面实施“七五”普法规划，推动形成尊法学法守法用法的良好社会氛围。

　　强化作风建设。深入学习习近平新时代中国特色社会主义思想，推进“两学一做”学习教育常态化制度化，精心组织开展“不忘初心、牢记使命”主题教育。深入落实中央八项规定实施细则，驰而不息整治“四风”。严格落实密切联系群众机制，深入基层、深入一线开展调查研究，拓展畅通民情民意表达渠道。健全抓落实长效机制，层层分解任务，加强督查督办，严格绩效考核，推动各项任务有效落实。

　　强化廉政建设。认真履行“一岗双责”，严守政治纪律、组织纪律、廉洁纪律、群众纪律、工作纪律和生活纪律。加强国有资产、资金、资源监管，完善预算编制和监督机制，强化行政监察和审计监督，扎牢制度笼子，营造干部清正、政府清廉、政治清明的良好氛围。

　　各位代表、各位委员，迈进新时代，开启新征程，肩负新使命。让我们更加紧密地团结在以习近平同志为核心的党中央周围，在省委、省政府和市委的坚强领导下，奋发进取，埋头苦干，加快建设动力之城、特色之城、生态之城、活力之城、和谐之城，全面开创新一轮振兴发展、决胜全面小康的崭新局面！
